# *Lactiplantibacillus plantarum* subsp. *plantarum* and Fructooligosaccharides Combination Inhibits the Growth, Adhesion, Invasion, and Virulence of *Listeria monocytogenes*

**DOI:** 10.3390/foods11020170

**Published:** 2022-01-10

**Authors:** Qingli Dong, Xinxin Lu, Binru Gao, Yangtai Liu, Muhammad Zohaib Aslam, Xiang Wang, Zhuosi Li

**Affiliations:** School of Health Science and Engineering, University of Shanghai for Science and Technology, Shanghai 200093, China; dongqingli@126.com (Q.D.); luxin_xin@163.com (X.L.); gao_binru@163.com (B.G.); usstlyt@163.com (Y.L.); zohaib.aslam.000@gmail.com (M.Z.A.); xiang.wang@usst.edu.cn (X.W.)

**Keywords:** *Listeria monocytogenes*, fructooligosaccharide, *Lactiplantibacillus plantarum* subsp. *plantarum*, human cells, gene expression

## Abstract

*Listeria monocytogenes* is a foodborne pathogen responsible for many food outbreaks worldwide. This study aimed to investigate the single and combined effect of fructooligosaccharides (FOS) and *Lactiplantibacillus plantarum* subsp. *plantarum* CICC 6257 (*L. plantarum*) on the growth, adhesion, invasion, and virulence of gene expressions of *Listeria monocytogenes* 19112 serotype 4b (*L. monocytogenes*). Results showed that *L. plantarum* combined with 2% and 4% (*w*/*v*) FOS significantly (*p* < 0.05) inhibited the growth of *L. monocytogenes* (3–3.5 log_10_ CFU/mL reduction) at the incubation temperature of 10 °C and 25 °C. Under the same combination condition, the invasion rates of *L. monocytogenes* to Caco-2 and BeWo cells were reduced more than 90% compared to the result of the untreated group. After *L. plantarum* was combined with the 2% and 4% (*w*/*v*) FOS treatment, the gene expression of actin-based motility, sigma factor, internalin A, internalin B, positive regulatory factor A, and listeriolysin O significantly (*p* < 0.05) were reduced over 91%, 77%, 92%, 89%, 79%, and 79% compared to the result of the untreated group, respectively. The inhibition level of the *L. plantarum* and FOS combination against *L. monocytogenes* was higher than that of FOS or *L. plantarum* alone. Overall, these results indicated that the *L. plantarum* and FOS combination might be an effective formula against *L. monocytogenes*.

## 1. Introduction

*Listeria monocytogenes* is a foodborne pathogen that causes a severely invasive disease called listeriosis. It can infect healthy individuals, but it most commonly affects immunocompromised individuals, pregnant women, newborns, and the elderly [1]. *L. monocytogenes* is ubiquitous in the environment and is resistant to environmental stresses, such as low temperature, low acid, and high osmolarity concentrations [2]. Due to these features, *L. monocytogenes* is considered a major concern for the food industry. The largest outbreak of listeriosis was reported in South Africa with the consumption of polony (ready-to-eat processed meat) in 2017. From 11 June 2017 to 7 April 2018, a staggering total of 937 cases were identified in that outbreak, of which 193 (27%) died, 465 (50%) were associated with pregnancy, and 406 of the pregnancy-associated cases (87%) occurred in newborn infections [3]. Although the infection rate per year is not high (such as in 2019, the European Union notification rate of 0.46 cases per 100,000 population was reported), the lethality is very high (20–30%) [4]. Therefore, it is necessary to strengthen the control of *L. monocytogenes* in foods.

In recent years, many chemical, physical, and biological technologies have been used as practical approaches for controlling *L. monocytogenes* in foods [4]. According to the International Scientific Association of Probiotics and Prebiotics (ISAPP), the definition of probiotics is “live micro-organisms benefiting the host” [5]. Among those technologies against *L. monocytogenes*, probiotics and their metabolites in foods are considered an effective biological control method that has drawn much attention from the scientific community. Several studies have reported that probiotics can inhibit the growth of *L. monocytogenes* [5,6,7]. Kamiloeglu et al. reported that the *L. monocytogenes* concentration decreased by 274 log_10_ CFU/g in the presence of *Lactiplantibacillus plantarum* S50 in fermented sausage [8]. Our previous study has also verified that *Lactiplantibacillus plantarum* subsp. *plantarum* CICC 6257 (*L. plantarum*) could effectively decrease the concentration of *L. monocytogenes* in ground pork [9]. Other bacteriocin-producing strains of lactic acid bacteria, including *Lactococcus lactis* ssp. *lactis*, have been found with a similar effect on *L. monocytogenes* (decreased by 2–3 log_10_ CFU/g) in different types of foods [10,11,12]. These antibacterial properties of probiotics on *L. monocytogenes* are mainly attributed to the production of antibacterial substances, such as bacteriocins, organic acids (lactic and acetic acid), hydrogen peroxide, and nutritional competition between probiotics and *L. monocytogenes* [12,13,14].

Many studies have reported that a combination of technologies and approaches (called hurdle technology) is more effective for controlling *L. monocytogenes* than single technology [15,16]. Prebiotics are “components that are selectively utilized by host microorganisms to confer a health benefit” according to ISAPP [17]. Based on the hurdle technology, the combination of probiotics and prebiotics has been reported to control the growth of enteric bacterial pathogens in vivo [18,19]. This phenomenon might be called competition enhancement, i.e., specific nutrients are given to symbiotic microorganisms in the same ecological niche, to better control the growth of pathogenic bacteria [20,21]. Fructooligosaccharides (FOS) are generally regarded as safe (GRAS) for human consumption [22], and a dose of 4~15 g/day given to healthy subjects [23] or patients with type 2 diabetes [22] does not show a side effect. FOS are present naturally in several fruits and vegetables [24], and is the most extensively studied prebiotic [25]. FOS could selectively stimulate *Lactobacillus* spp. or *Bifidobacterium* spp. [26,27,28], and inhibit *Escherichia coli* in vivo [29]. In vitro, FOS were found to inhibit the growth of *Pseudomonas aeruginosa* [30] and *Salmonella typhimurium* [31] in the culture medium, and reduce the adhesion of *Clostridium difficile* [32] and some anaerobic pathogens [33] to host cells. The combined treatment of *Lactobacillus rhamnosus NCDC 298* and FOS prevented the adhesion of enterotoxigenic *E. coli* to HT-29 cells [34]. Combined *Lactobacillus brevis* KU200019 and FOS inhibited pathogen adherence to HT-29 cells [35]. Thus, in this study, we use FOS and *L. plantarum* as a hurdle technology to explore its possible inhibitory effect on *L. monocytogenes.*

After ingesting contaminated food, *L. monocytogenes* could cross the intestinal epithelial barrier into the lamina propria, disseminate into blood, and even cross the blood–brain barrier or the placental barrier, which are the tightest barriers of the human body [36]. The adhesion and invasion ability of the pathogen to the host cell is an important and useful approach to judge the effects of bacteriostatic measures [37]. Specifically, the mild biological bacteriostatic method often could not eliminate bacteria; thus, it becomes more critical to evaluate the virulence of bacteria in vitro or in vivo after biological control. Caco-2 cells [38,39] and BeWo cells [40,41], as the most commonly used representatives of intestinal and fetoplacental barrier models, respectively, were used to evaluate the adhesion and invasion of foodborne pathogens in vitro. It has been proven that *L. monocytogenes* can adhere and invade Caco-2 cells [42,43] and BeWo cells [44,45].

*L. monocytogenes* is a facultative intracellular pathogen that infects phagocytes and normally non-phagocytic cells, such as epithelial cells [46]. It can resist the adverse environment of the gastrointestinal tract, survive, and divide in the cytosol of host cells, and spread from one cell to another [46,47,48]. These processes occur in several stages and require an elaborate network of virulent factors, among which sigma factor (*sigB*), positive regulatory factor A (*prfA*), internalin A (*inlA*), internalin B (*inlB*), actin-based motility (*actA)*, and listeriolysin O (*hly*) are considered the principal determinants. When *L. monocytogenes* infects host cells, *sigB* is involved in the stress response, regulating many stress-related genes [49]. The *inlA* and *inlB* play core roles in internalizing *L. monocytogenes* into host cells [50]. After internalization, *L. monocytogenes* produces listeriolysin O (*hly*), which mediates the escape of *L. monocytogenes* from phagosomes [51]. Meanwhile, *actA* is involved in the adhesion and intracellular motility of *L. monocytogenes* [52]. As mentioned above, the action of virulence factors contributes to *L. monocytogenes* infection. To better prove the effect of FOS and *L. plantarum*, the virulence factors of *L. monocytogenes* should also be evaluated.

Therefore, in this study, we tried to explore the single or joint effect of *L. plantarum* and FOS on *L. monocytogenes* potential growth, its adhesion and invasion to Caco-2 and BeWo cells, and its virulence genes expression.

## 2. Materials and Methods

### 2.1. Bacterial Strains and Culture Preparation

The reference strains *L. monocytogenes* 19112 (serotype 4b) and the strain *L. plantarum* CICC 6257 were obtained from the China Center of Industrial Culture Collection Beijing (CICC, http://www.china-cicc.org/, accessed on 11 September 2021). Frozen stocks of *L. monocytogenes* were maintained in tryptone soy yeast extract broth (TSB-YE; Beijing Land Bridge Technology Co., Ltd., Beijing, China) with 50% glycerol at −80 °C. Working stocks of *L. monocytogenes* were stored at 4 °C on tryptone soy agar with 0.6% yeast extract (TSA-YE; Beijing Land Bridge Technology Co., Ltd., Beijing, China) and were renewed monthly. Frozen stocks of *L. plantarum* were maintained in MRS broth (MRSB, Hopebio, Qingdao, China) with 50% glycerol at −80 °C. Working stocks of *L. plantarum* were stored at 4 °C on MRS agar (MRSA, Hopebio, Qingdao, China) and were renewed monthly. For activation, a single colony of *L. monocytogenes* and *L. plantarum* were separately transferred from TSA-YE and MRS to the brain heart infusion broth (BHI, Beijing Luqiao Co., Beijing, China), and aerobically incubated at 37 °C for 16~18 h. After that, the *L. monocytogenes* and *L. plantarum* cultures were centrifuged at 21,127× *g*/min for 10 min (4 °C), washed thrice, and then resuspended in 0.85% sterile saline solution (SSS, pH = 7.2) to 10^8^–10^9^ CFU/mL inoculums.

### 2.2. Sample Preparation and Inoculation

It is reported that 2% FOS added in yogurt showed an inhibitory effect on *L.monocytogenes* [35], and did not show adverse sensory properties [53]. We found that less than 1% FOS treatment did not show an inhibitory effect on *L. monocytogenes*. Thus, based on previous reports and our preliminary studies, 1~4% FOS was used for evaluating the effect of FOS. *L. monocytogenes* was diluted to inoculate (10^2^–10^3^ CFU/mL) in BHI with or without 1~4% (*w*/*v*) FOS (Shanghai yuanye Bio-Technology Co., Ltd., CAS: 308066-66-2, purity 95%, Shanghai, China) and incubated at 10 °C and 25 °C. At 10 °C, *L. monocytogenes* is in the growth phase during the first 10 days of incubation, thus the sampling was performed once a day; while after 10 days, the *L. monocytogenes* is in a stationary phase, so the sampling was performed every two days. Like 10 °C, at 25 °C every four hours before 24 h of incubation and a longer interval after 24 h were set as the sampling timelines. Thus, sampling was performed on days 0, 2, 3, 4, 5, 6, 7, 8, 10, 12, 14, 16, and 18 at 10 °C, and on 0, 5, 9, 13, 17, 21, 27, and 45 h at 25 °C until inhibitory effects were observed. The apparent inhibitory effect of the FOS alone treatment was observed after 18 days at 10 °C and 45 h at 25 °C; thus, this incubation condition was used in the subsequent experiment.

To evaluate the combined effect of *L. plantarum* and FOS, both *L. monocytogenes* and *L. plantarum* were diluted to 10^2^–10^3^ CFU/mL in BHI with or without 1~4% (*w*/*v*) FOS and incubated for 18 days at 10 °C and 45 h at 25 °C, respectively. Thus, the treatment of *L. monocytogenes* was divided into eight groups: the untreated group, 1% FOS, 2% FOS, 4% FOS, *L. plantarum* alone, *L. plantarum* + 1% FOS, *L. plantarum* + 2% FOS, and *L. plantarum* + 4% FOS. The *L. monocytogenes* and *L. plantarum* concentrations were determined by plating on the PALCAM (Polymyxin Acriflavin Lithium-chloride Ceftazidime Esculin Mannitol) agar base with selective supplement (PALCAM, Qingdao Haibo Co., Ltd., Qingdao, China) and MRSA, respectively, followed by aerobic incubation at 37 °C for 24 h [9]. The counts of colonies were expressed as log_10_ CFU/mL.

### 2.3. In Vitro Virulence Assays

The human intestinal epithelial Caco-2 cells (FH0029, passage 10~20) were obtained from FuHeng Biology (Shanghai, China) and cultured in Dulbecco’s modified Eagle medium (DMEM) containing 10% fetal bovine serum (FBS), 1.25% L-glutamine, and 1.25% penicillin-stretomycin solution in a humidified atmosphere of 95% air and 5% CO_2_ at 37 °C. The choriocarcinoma cell line BeWo (FH0248, passage 5~10) obtained from FuHeng Biology were cultured in Ham’s F12 medium Cellgro (www.cellgro.com, accessed on 11 September 2021) with 10% FBS, and 1.25% penicillin-streptomycin solution in a humidified atmosphere of 95% air and 5% CO_2_ at 37 °C.

The adhesion and invasion of *L. monocytogenes* to Caco-2 cell monolayers were assessed as described by the previous study [37,54]. Briefly, Caco-2 cells were plated into 12-well tissue culture plates (Greiner Bio-One, Frickenhausen, Germany) in DMEM with 10% FBS and 1.25% penicillin-streptomycin solution and incubated until 90% confluence.

*L. monocytogenes* with and without FOS and *L. plantarum*, were cultivated at 10 °C for 12 days and 25 °C for 40 h. These bacterial cultures were centrifuged (5282× *g*/min, 5 min), and resuspended in pre-heated DMEM without FBS and penicillin-stretomycin solution at 37 °C. The initial *L. monocytogenes* counting was performed by plating on PALCAM and denoted as *N*_0_. For adhesion, Caco-2 and BeWo cell monolayers were infected with *L. monocytogenes* cultures described above for 2 h at 37 °C. Caco-2 and BeWo cells were then washed twice with phosphate-buffered saline (PBS) and lysed with 1 mL of cold 0.1% Triton X-100 (Applichem, Darmstadt, Germany) for 5 min. After adhesion, the *L. monocytogenes* counting was performed by plating on PALCAM and denoted as *N*_1_.

For the invasion assay, Caco-2 and BeWo cell monolayers were infected with *L. monocytogenes* cultures for 2 h at 37 °C as described above. Cells were then washed twice with PBS, incubated in DMEM containing 0.1% FBS and 100 µg/mL penicillin-streptomycin for 45 min and lysed with 1 mL of cold 0.1% Triton X-100 for 5 min. After adhesion, the *L. monocytogenes* counting was performed by plating on PALCAM and denoted as *N*_2_. All assays were independently repeated six times. Most previous reports used 1–2 h to detect the in vitro adhesion and invasion [55], so we used 1, 1.5, and 2 h for evaluation, and 2 h was the shortest time to detect the difference among different treatment groups.

The adhesion and invasion efficiencies were calculated as the following equations:

$$\mathrm{Adhesion}\;\mathrm{efficiency}=N_{1}/N_{0}\times 100\%$$

$$\mathrm{Invasion}\;\mathrm{efficiency}=N_{2}/N_{0}\times 100\%$$

While *N*_0_ is the number of *L. monocytogenes* in the initial inoculum; *N*_1_ is the number of *L. monocytogenes* that adhered to cells; and *N*_2_ is the number of *L. monocytogenes* that invaded into cells.

### 2.4. Analysis of Virulence Factors of L. monocytogenes by Quantitative Real-Time PCR (qRT-PCR)

*L. monocytogenes* with and without treatment with FOS or *L. plantarum*, cultivated at 10 °C for 12 days and 25 °C for 40 h, was separately applied for total RNA extraction. The total RNA was extracted using the Bacteria Total RNA Isolation Kit (Sangon Biotech, Shanghai, China) based on a standard protocol and quantified by a NanoDrop 2000 (Thermo Fisher Scientific, Waltham, MA, USA). Subsequently, the residual DNA was removed from the total RNA and the cDNA was synthesized, using Hiscript^®^ II Reverse Transcriptase Kit (Vazyme Biotech Co., Ltd., Nanjing, China). Primers used in this study were designed by Zilelidou et al. [55], synthesized by Sangon Biotech Co., Ltd. (Shanghai, China), and listed in Table 1. An analysis of qRT-PCR was performed using the ABI 7900HT real-time PCR system (Applied Biosystems, Foster, USA) in 20 μL reaction solution, including 1.2 μL cDNA template (ca. 120 ng), total 10 μL Taq Pro Universal SYBR qPCR Master Mix (2×), 8 μL nuclease-free water, and 0.8 μL primer (10 μM). The intergenic spacers *rpoB* were used as housekeeping genes; *prfA*, *sigB*, *hly*, *actA*, *inlA*, and *inlB* as virulence factors of *L. monocytogenes* were evaluated as well. The amplification program for RT-qPCR was as follows: 1 cycle at 95 °C for 30 s, 40 cycles at 95 °C for 5 s, and 60 °C for 30 s; a melt curve program, 95 °C for 10 s, 65 °C for 60 s, and 97 °C for 1 s, was added to evaluate the specificity of the RT-PCR products. The relative transcription level of the five virulent genes, *sigB*, *inlA*, *inlB*, *hly*, and *prfA*, was calculated using the 2^−ΔΔCt^ method [56].

### 2.5. Statistical Analysis

All assays were performed in at least six biological independent experiments, and the results were shown as mean values with standard deviations. The ANOVA analysis (SPSS 18.0 statistical software, IBM Corporation, Armonk, NY, USA) followed by a Tukey test at 95% confidence limits were applied to determine the difference in the growth, the ability of adhesion and invasion to the cell, and the relative expression of the virulence genes of *L. monocytogenes*.

## 3. Results

### 3.1. Effectiveness of FOS Treatment against L. monocytogenes in BHI

**Figure 1:**
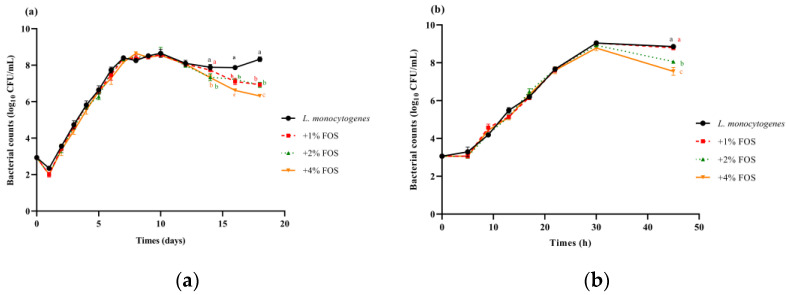
Growth kinetics of *L. monocytogenes* with the presence of 1% (*w*/*v*) FOS, 2% (*w*/*v*) FOS, or 4% (*w*/*v*) FOS in BHI at 10 °C for 18 days (**a**) and at 25 °C for 45 h (**b**). Data represented as log_10_ (CFU/mL) are mean values ± SD with (*n* = 6).

The *L. monocytogenes* concentration in the untreated group increased to day 10 at 10 °C, and an increasing trend was observed on days 16–18 (Figure 1a). After 16 days, *L. monocytogenes* concentrations in the FOS treatment groups were significantly decreased compared with the untreated group at 10 °C (Figure 1a). Among them, *L. monocytogenes* concentrations in the 1%, 2%, and 4% FOS groups showed the reduction of 1, 1, and 2 log_10_ CFU/mL compared with the untreated group on day 18 at 10 °C, respectively (Figure 1a). After 27 h at 25 °C, *L. monocytogenes* concentrations in the 2% and 4% FOS treatment groups significantly decreased compared with the untreated group at 25 °C (Figure 1b). Among them, *L. monocytogenes* concentrations in the 1%, 2%, and 4% FOS groups represent the reduction of 0.1, 0.8, and 1.5 log_10_ CFU/mL compared with the untreated group at 45 h at 25 °C (Figure 1b). It was observed that FOS treatments against *L. monocytogenes* were more effective at 10 °C as compared to 25 °C (Figure 1a,b).

### 3.2. Effectiveness of L. plantarum and FOS Combination Treatment against L. monocytogenes in BHI

**Figure 2:**
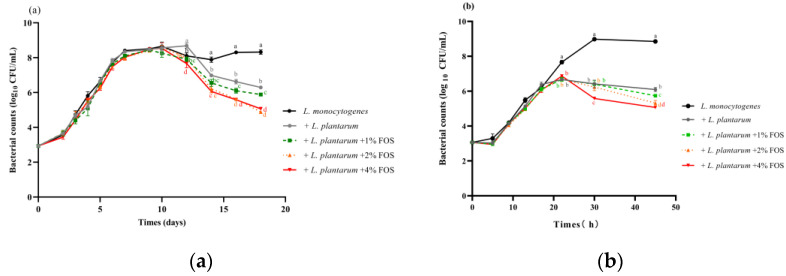
Growth kinetics of *L. monocytogenes* on *L. plantarum* combined with 1% (*w*/*v*) FOS, 2% (*w*/*v*) FOS, or 4% (*w*/*v*) FOS in BHI at 10 °C for 18 days (**a**) and at 25 °C for 45 h (**b**). Data represented as log_10_ (CFU/mL) are mean values ± SD (*n* = 6).

The effect of the *L. plantarum* and FOS combination on the growth of *L. monocytogenes* at 10 °C and 25 °C was investigated. When *L. monocytogenes* was incubated with *L. plantarum* at 10 °C, the growth of *L. monocytogenes* was significantly (*p* < 0.05) reduced after 10 days compared with the untreated group (Figure 2a). The *L. plantarum* and FOS combination treatment showed a more obvious inhibitory effect than the *L. plantarum* alone or FOS alone treatment (Figure 2a). *L. monocytogenes* concentrations in *L. plantarum*, *L. plantarum* + 1% FOS, *L. plantarum* + 2% FOS, and *L. plantarum* + 4% FOS groups represented a reduction of 2, 2.5, 3, and 3 log_10_ CFU/mL compared with the untreated group on 18 days at 10 °C (Figure 2a).

At 25 °C, the addition of *L. plantarum* significantly (*p* < 0.05) inhibited the growth of *L. monocytogenes* compared with the untreated group after 21 h of storage (Figure 2b). *L. monocytogenes* concentrations with *L. plantarum* alone, *L. plantarum* + 1% FOS, *L. plantarum* + 2% FOS, and *L. plantarum* + 4% FOS treatment represented a reduction of 2.5, 3, 3.5, and 3.5 log_10_ CFU/mL compared with the untreated group at 45 h at 25 °C (Figure 2b). Similarly, the *L. plantarum* and FOS combination treatment showed a more obviously inhibited effect on the growth of *L. monocytogenes* than the *L. plantarum* alone or FOS alone treatment at 25 °C (Figure 2b).

### 3.3. Ability of Adhesion and Invasion of L. monocytogenes to Caco-2 and BeWo Cells In Vitro

**Figure 3:**
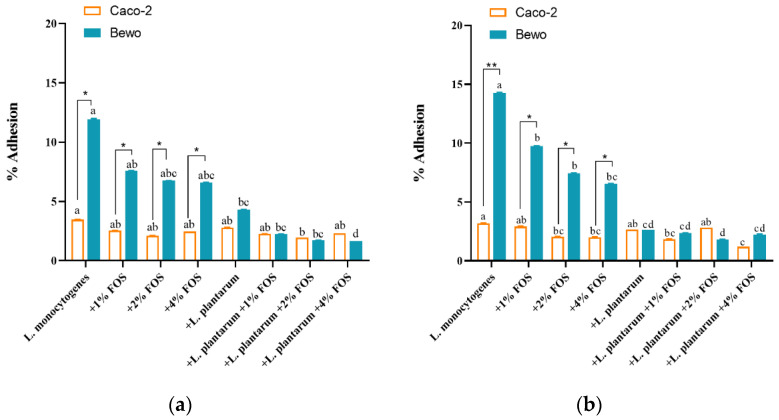
Adhesion to Caco-2 cells and BeWo cells of *L. monocytogenes*, grown in BHI at 10 °C for 12 days (**a**) or at 25 °C for 40 h (**b**) in different treatment groups: (1) the untreated, (2) 1% (*w*/*v*) FOS, (3) 2% (*w*/*v*) FOS, (4) 4% (*w*/*v*) FOS, (5) *L. plantarum* supplementation, (6) *L. plantarum* + 1% (*w*/*v*) FOS, (7) *L. plantarum* + 2% (*w*/*v*) FOS, (8) *L. plantarum* + 4% (*w*/*v*) FOS groups. Values are mean ± SD (*n* = 6). Asterisks indicate significant differences between the invasion rate to Caco-2 and BeWo cells, * *p* < 0.05, ** *p* < 0.01. Different lowercase letters (a, b, c, and d) indicate significant differences (*p* < 0.05) among treatments for each cell lines.

Then we investigated the single or joint effect of *L. plantarum* and FOS on in vitro adhesion and invasion of *L. monocytogenes* to Caco-2 and BeWo cells. The adhesion and invasion of *L. monocytogenes* were measured after incubation at 10 °C for 12 days and 25 °C for 40 h, respectively. At 10 and 25 °C, the adhesion rates of *L. monocytogenes* to BeWo cells were significantly higher than those of Caco-2 cells in the untreated or FOS treatment groups (Figure 3a,b). Additionally, the invasion rate of *L. monocytogenes* to BeWo cells in all eight groups at 25 °C for 40 h was higher than that of Caco-2.

At 10 °C, for the Caco-2 and BeWo cells, different concentrations of FOS had no significant (*p* > 0.05) inhibitory effect on the adhesion rate of *L. monocytogenes* compared with the untreated group (Figure 3a). However, the combined treatment of *L. plantarum* + FOS significantly (*p* < 0.05) reduced the adhesion rate of *L. monocytogenes* in BeWo cells (Figure 3a). Specifically, the adhesion rate of *L. monocytogenes* to BeWo cells was the lowest in *L. plantarum* + 4% FOS (Figure 3a). The adhesion rate of *L. monocytogenes* to Caco-2 cells was significantly reduced by the *L. plantarum* + 2% FOS treatment compared with the untreated group (Figure 3a). At 25 °C, the adhesion rates of *L. monocytogenes* to Caco-2 cells were significantly (*p* < 0.05) reduced in the 2% FOS, 4% FOS, *L. plantarum* + 1% FOS, and *L. plantarum* + 4% FOS groups compared with the untreated group (Figure 3b). All treatment conditions significantly reduced the adhesion rates of *L. monocytogenes* to BeWo cells (Figure 3b).

**Figure 4:**
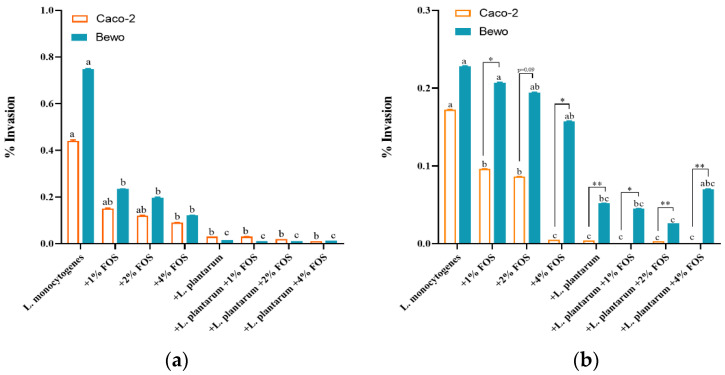
Invasion to Caco-2 cells and BeWo cells of *L. monocytogenes* grown in BHI at 10 °C for 12 days (**a**) or at 25 °C for 40 h (**b**) in different treatment groups: (1) the untreated, (2) 1% (*w*/*v*) FOS, (3) 2% (*w*/*v*) FOS, (4) 4% (*w*/*v*) FOS, (5) *L. plantarum* supplementation, (6) *L. plantarum* + 1% (*w*/*v*) FOS, (7) *L. plantarum* + 2% (*w*/*v*) FOS, (8) *L. plantarum* + 4% (*w*/*v*) FOS groups. Values are mean ± SD (*n* = 6). Asterisks indicate significant differences between the invasion rate to Caco-2 and BeWo cells, * *p* < 0.05, ** *p* < 0.01. Different lowercase letters (a, b, and c) indicate significant differences (*p* < 0.05) among treatments for each cell lines.

At 10 °C, all treatment significantly reduced the invasion rates of *L. monocytogenes* to BeWo cells. After the combined treatment of *L. plantarum* and FOS, over 90% of the invasion levels were reduced compared to the result of the untreated group (Figure 4a). At 10 °C, the invasion rates of *L. monocytogenes* to Caco-2 cells were significantly (*p* < 0.05) reduced in the 4% FOS, *L. plantarum*, *L. plantarum* + 1% FOS, *L. plantarum* + 2% FOS, and *L. plantarum* + 4% FOS groups compared with the results of the untreated group (Figure 4a). At 25 °C, the invasion rate of *L. monocytogenes* to Caco-2 were significantly (*p* < 0.05) reduced to less than 0.001% using treatments of 4% FOS, *L. plantarum*, *L. plantarum* + 1% FOS, *L. plantarum* + 2% FOS, and *L. plantarum* + 4% FOS (Figure 4b). For BeWo cells, the *L. plantarum* addition treatment significantly reduced the invasion rate of *L. monocytogenes* at 10 °C (Figure 4b). In addition, at 25 °C the invasion rates of *L. monocytogenes* to BeWo cells were significantly higher than those of Caco-2 cells in different treatment groups (Figure 4b); similar results were not found at 10 °C.

### 3.4. Virulence Genes Expression of L. monocytogenes

**Figure 5:**
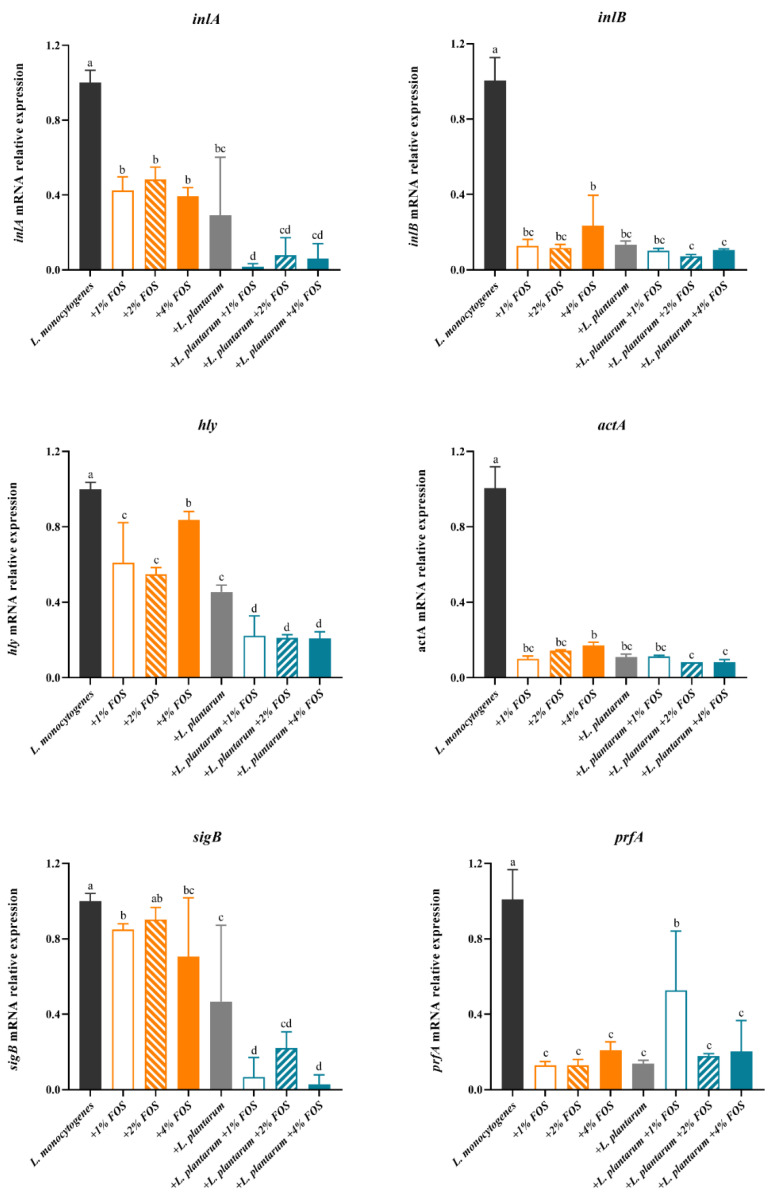
Relative expression levels of virulence genes of *L. monocytogenes* in BHI at 10 °C for 12 days in different treatment groups: (1) the untreated group (black bar), (2) 1% (*w*/*v*) FOS (orange hollow bars), (3) 2% (*w*/*v*) FOS (slash orange bars), (4) 4% (*w*/*v*) FOS (orange bars), (5) *L. plantarum* supplementation group (dark gray bars), (6) *L. plantarum* + 1% (*w*/*v*) FOS (blue hollow bars), (7) *L. plantarum* + 2% (*w*/*v*) FOS (slash blue bars), (8) *L. plantarum* + 4% (*w*/*v*) FOS (blue bars). Values are mean ± SD (*n* = 6). At each temperature, different lowercase letters (a, b, c, and d) indicate significant differences (*p* < 0.05) among treatments.

We investigated the transcriptional profiles of key *L. monocytogenes* virulence genes associated with invasion and intracellular proliferation into host cells. The expression levels of *inlA*, *inlB*, *sigB*, *prfA*, *hly*, and *actA* in *L. monocytogenes* at 25 °C for 40 h and at 10 °C for 12 days were determined. At 10 °C for 12 days, *inlA*, *inlB*, *hly*, *actA*, *sigB*, and *prfA* genes expression of *L. monocytogenes* were significantly decreased by FOS alone, *L. plantarum* alone, and FOS + *L. plantarum* combined treatment, compared with that of the untreated group (Figure 5). At 10 °C for 12 days, *inlA*, *hly*, and *sigB* genes expression of *L. monocytogenes* in the FOS and *L. plantarum* combination group displayed to be obviously inhibited compared with the FOS or *L. plantarum* alone treatment groups. In contrast, no significant differences in *inlB*, *prfA*, and *actA* gene expression were observed among FOS alone, *L. plantarum* alone, and FOS + *L. plantarum* combination groups (Figure 5). Furthermore, the gene expression of *prfA* at 10 °C with 1% FOS + *L. plantarum* treatment did not show similar results compared with that of 1% FOS treatment.

**Figure 6:**
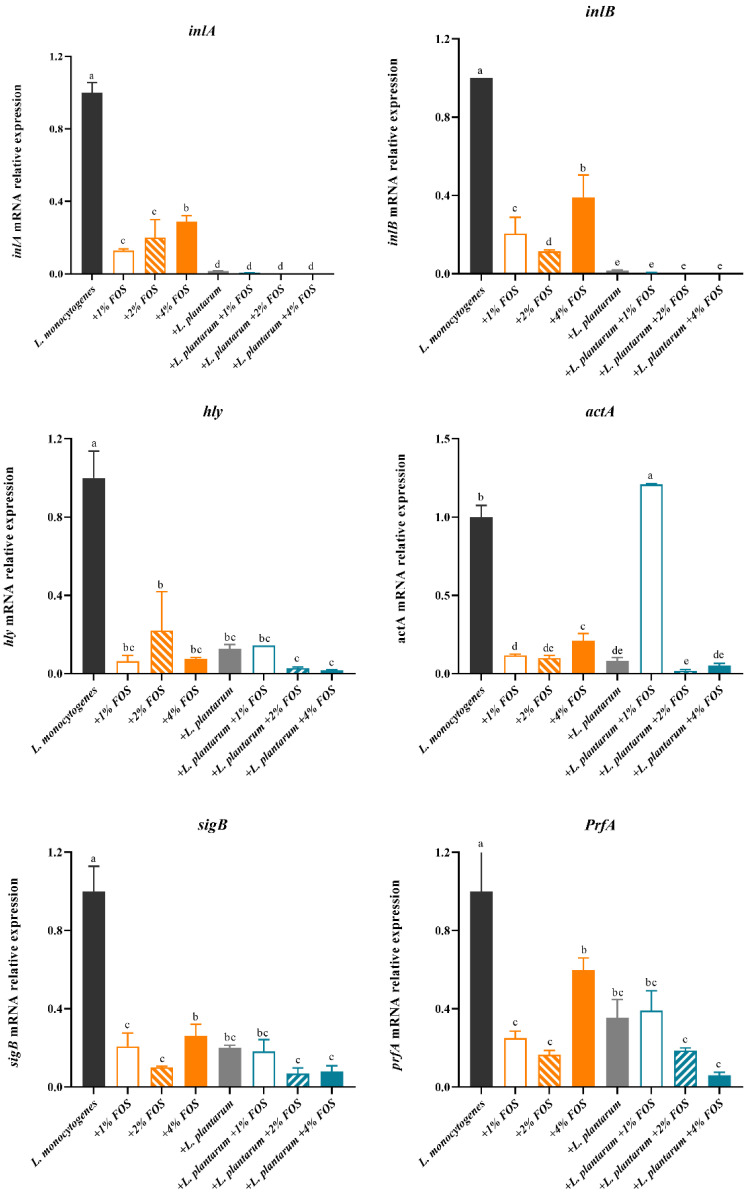
Relative expression levels of virulence genes of *L. monocytogenes* in BHI at 25 °C for 40 h in different treatment groups: (1) the untreated group (black bar), (2) 1% (*w*/*v*) FOS (orange hollow bars), (3) 2% (*w*/*v*) FOS (slash orange bars), (4) 4% (*w*/*v*) FOS (orange bars), (5) *L. plantarum* supplementation group (dark gray bars), (6) *L. plantarum* + 1% (*w*/*v*) FOS (blue hollow bars), (7) *L. plantarum* + 2% (*w*/*v*) FOS (slash blue bars), (8) *L. plantarum* + 4% (*w*/*v*) FOS (blue bars). Values are mean ± SD (*n* = 6). At each temperature, different lowercase letters (a, b, c, d, and e) indicate significant differences (*p* < 0.05) among treatments.

At 25 °C for 40 h, *inlA*, *inlB*, *hly*, *sigB*, and *prfA* genes expression of *L. monocytogenes* were significantly decreased by the FOS, *L. plantarum*, and FOS + *L. plantarum* treatment, compared with the results of the untreated group (Figure 6). At 25 °C for 40 h compared with the FOS alone treatment, the *L. plantarum* addition could significantly (*p* < 0.05) reduce the expression of *inlA* and *inlB* genes. The gene expressions of *inlA* and *inlB* in the *L. plantarum* and FOS combination groups were about 90% lower than that of the untreated group (Figure 6). The expression levels of *hly*, *PrfA*, and *sigB* genes in the FOS alone, *L. plantarum* alone, and FOS and *L. plantarum* combination groups were about 40%-85% lower than that of the untreated group (Figure 6). The expression levels of the *actA* gene were significantly reduced in all treatment groups except the *L. plantarum* + 1% FOS group compared with that of the untreated group (Figure 6). The *L. plantarum* + 1% FOS treatment showed an inhibitory effect on the growth, adhesion, and invasion of *L. monocytogenes*, but a poor inhibitory effect on *actA* and *PrefA* gene expressions, compared with the 1% FOS treatment. The FOS was freshly prepared before each use, and the other concentration of FOS treatment showed an inhibitory effect (Figure 6). Thus, we speculated that the addition of *L. plantarum* may affect the action of 1% FOS, extended processing time will be helpful to verify our hypothesis in the future.

## 4. Discussion

In the present study, we examined the single and combined effect of FOS and *L. plantarum* on the growth and in vitro virulence of *L. monocytogenes*. We observed that *L. plantarum* alone or FOS + *L. plantarum* effectively reduced the growth of *L. monocytogenes* during storage at 10 °C and 25 °C. Furthermore, the FOS, *L. plantarum*, or FOS + *L. plantarum* treatment also decreased the invasion of *L. monocytogenes* to Caco-2 and BeWo cells, and down-regulated the expression levels of the *inlA*, *inlB*, *hly*, *actA*, *sigB*, and *prfA* genes of *L. monocytogenes*, which play key roles in the infection process of *L. monocytogenes*.

In this study, 10 °C and 25 °C simulated the room or refrigerated temperature at which food is processed and stored. The growth of *L. monocytogenes* at 37 °C was also investigated (data not shown); there was no difference between treatment groups. We consider that this is because 37 °C is the most suitable temperature for the growth of *L. monocytogenes;* therefore, addition changes have little effect on its growth at 37 °C. Thus, in this study, we set the process that food contaminated with *L. monocytogenes* was placed at 10 °C and 25 °C for a period, and ingested by a human, followed by *L. monocytogenes* infecting host cells at 37 °C.

Probiotics can inactivate pathogens via several mechanisms, including the production of antimicrobial substances, competition of nutrients, and cell-to-cell contact [57,58]. According to the strain instruction manual, the strain *L. plantarum* CICC 6257 used in this study is recommended as a probiotic and has an inhibitory effect against several bacteria. However, no related report introduces its probiotic function and antibacterial effect in vivo or in vitro. We did not investigate the in vivo probiotic activity of this strain, while our previous studies reported its in vitro inhibitory effect on *L. monocytogenes* in ground pork [9,15]. Except for the strain *L. plantarum* CICC 6257, the other *Lacticaseibacillus rhamnosus* strain was used but did not show an inhibitory effect against *L. monocytogenes* (data not shown). Thus, in this study, only this strain *L. plantarum* CICC 6257 was selected for exploring an inhibitory effect against *L. monocytogenes* in the simulated food substrate. In the future, it is necessary to compare more different probiotic strains to validate our results.

Our study showed that the co-culture of *L. plantarum* caused the *L. monocytogenes* concentration to decrease over 2 log_10_ CFU/mL. The growth of *L. monocytogenes* was reduced only when *L. plantarum* accumulated to enough concentration (> 8 log_10_ CFU/mL) at 10 °C and 25 °C. Similar results have been reported, such as *Lactobacillus curvatus* reducing the growth of *L. monocytogenes* when *L. curvatus* reached 9 log_10_ CFU/mL after 48 h at 20 °C and 5 days at 15 °C [59]. This might be the result of nutrients competition between *L. plantarum* and *L. monocytogenes*, or attributed to inhibitory effect of metabolites (organic acids, diacetyl, hydrogen peroxide, and bacteriocins) produced by *L. plantarum* [60]. Schillinger et al. reported that the inhibition of *L. monocytogenes* was principally attributed to the antibacterial action of bacteriocins produced by *Lactobacillus sake* [10]. Huang et al. [14] reported bacteriocin-producing *Enterococcus faecium* reduced the cell counts of *L. monocytogenes* after 4 days at 4 °C. Han et al. [61] also showed the bacteriocin-producing strain *Lactococcus lactis* KC24 reduced the cell counts of *L. monocytogenes* after 4 h at 35 °C. Thus, in this study the inhibitory effect of *L. plantarum* on *L. monocytogenes* growth occurred at 25 °C after 45 h and at 10 °C after 18 days, which might also be associated with the accumulation of bacteriocins produced by *L. plantarum* in the stationary phase. The mechanism of action of *L. plantarum*, including products of *L. plantarum*, needs to be designed and further studied in the future. In short, when *L. plantarum* reached a stable phase for a time, it could inhibit the growth of *L. monocytogenes*.

The combination of probiotics and prebiotics methods has been adopted to achieve a better inhibitory effect on pathogens in vivo and in vitro, such as how the combination of inulin/palatinose hydrate/α-cyclodextrin and *Lactobacillus* sp. or *Lactococcus* sp. strains could inhibit *L. monocytogenes* ATCC 19117 [20]. The combination of FOS and *L. brevis* KU200019 more effectively inhibited the adherence of *L. monocytogenes* ATCC 15313 and *Escherichia coli* O157:H4 FRIK 125 to HT-29 cells than FOS alone [35]. The combined treatment of chitosan and *Pediococcus acidilactici* was also found to inhibit the growth of *L. monocytogenes* in meatballs better than the single treatment by Incili et al. [62]. Similar to these previous reports, we also found that *L. plantarum* combined with 4% FOS had the best inhibitory effect on *L. monocytogenes*, and the *L. monocytogenes* concentration was reduced over 3 log_10_ CFU/mL at 25 °C for 45 h and 10 °C for 18 days. In short, the combined use of FOS and *L. plantarum* is more effective against *L. monocytogenes* growth than the single use.

There are conflicting conclusions about the effect of FOS on probiotics. FOS could act in different ways on probiotics [63], such as enhancing their bioactivity towards pathogens, increasing the production of bacteriocins, improving the growth rate, and decreasing the death rate [35,56,64]. In contrast, Lu et al. found that in an aerobic condition, the growth of *Lactobacillus* spp. and *Bifidobacterium* spp. strains was not promoted by FOS regardless of the carbohydrate source [64]. Our data showed that FOS did not accelerate the growth of *L.* *plantarum* (data not shown). Here, the ingredient of BHI needs to be considered. The BHI medium is rich in nutrients and contains beef heart extract, proteose peptone, glucose, sodium chloride, and disodium phosphate, all of which affect the growth and virulence of bacteria. We speculated *L.* *plantarum* preferentially used glucose of the BHI medium as a carbon source, and did not use FOS efficiently, and thus FOS did not promote the growth of *L.* *plantarum.* The investigation using other medium without carbon sources also need to be carried out in the future. Therefore, we speculate that the combined effect of FOS and *L. plantarum* against *L. monocytogenes* in the BHI culture system might not be due to the promotion of FOS to *L. plantarum*, but due to the additional inhibitory effect of FOS and *L. plantarum*.

Furthermore, it is worth noting that the BHI medium does not simulate any kind of food. The actual food system is often complicated due to differences and inconsistencies in food ingredients and background bacteria; however, the BHI medium is fixed and repeatable in composition and ideal for the growth of both *L. plantarum* and *L. monocytogenes*. Commercial mediums are used in many bacteriostatic or bactericidal studies [34,35,65]. Thus, in this study, the BHI medium as a testing substrate was used to judge the role of FOS and *L. plantarum* and ensure the repeatability of the experiment. The results in the BHI medium will provide a reference for performing a similar study on real food in the future.

Moreover, compared with the FOS and *L. plantarum* combined treatment, the treatment of FOS alone showed a relatively weaker inhibitory effect on the growth, adhesion, invasion, and virulence of gene expressions. Even so, considering FOS could be used as sugar substitutes and is easy to operate, it may be a better choice than the FOS and *L. plantarum* combination for controlling the *L. monocytogenes* in actual food production. The 4% FOS showed a reduction of 2 log_10_ CFU/mL compared with the control group at 10 °C. The inhibitory effect of the 4% FOS is similar to that of epsilon-polylysine [66] and nisin [65,67], which have been widely used as biological bacteriostatic agents in food. It is important to investigate the effect of FOS in real food in the future.

The adhesion and invasion abilities of the *L. monocytogenes* to host cells were investigated. The adhesion and invasion abilities of *L. monocytogenes* to Caco-2 cells are reduced when *L. monocytogenes* is co-cultured with *Lactobacillus* spp. [68], *Lactobacillus rhamnoides* [69], and *L. plantarum* [55]. Moroni et al. observed that the adhesion and invasion ability of *L. monocytogenes* to colonocytes was reduced by *Lactobacillus* [70]. Similarly, our results showed that supplementation of *L. plantarum* significantly reduced the invasion of *L. monocytogenes* to Caco-2 cells, although it did not change its adhesion. Only the 4% FOS combined with *L. plantarum* significantly (*p* < 0.05) inhibited the adhesion of *L. monocytogenes* to Caco-2 at 25 °C, but not at 10 °C. Chen et al. reported that human milk oligosaccharide treatment reduced the infection rate of *L. monocytogenes* to Caco-2 cells by 50% [71]. The FOS or *L. plantarum* treatment did not change the adhesion of *L. monocytogenes* to Caco-2 cells; however, its invasion ability was significantly lowered by more than 90%. These results showed that FOS, *L. plantarum*, or their combination effectively prevent *L. monocytogenes* from invading intestinal barrier.

Pregnant women are a susceptible subgroup of *L. monocytogenes* due to the adaptability, capacity to cross various host barriers, and unique intracellular lifestyle of *L. monocytogenes* [36], and possible exacerbation of critical immune tolerance mechanisms at the maternal–fetal interface in late pregnancy [72]. The BeWo cell line has been considered a useful in vitro placental barrier model for studying adhesion, infection [73], and transport [40]. Faralla et al. reported that in pregnant guinea pigs and mice, the virulence factor action endows *L. monocytogenes* with a strong invasion tendency to the placenta, but *L. monocytogenes* has little influence on other organs [74]. Our research confirmed this view and showed that *L. monocytogenes* had a higher adhesion and invasion ability to BeWo cells than Caco-2 cells. In addition, different from the result in Caco-2 cells, FOS, *L. plantarum*, or their combination displayed a stronger inhibited ability in both adhesion and invasion to BeWo cells. This is because *L. monocytogenes* infects Caco-2 and BeWo cells based on different infection mechanisms, for example, the virulence protein of *L. monocytogenes* acts on different acceptor proteins of the two cell lines [36]. Moreover, *L. monocytogenes* could destroy the barrier and enter into the host cells. The phase-contrast images of cells in the different treatment groups were observed; however, the difference among these images was not found (data not shown). In the future, to explore if *L. monocytogenes* influences the barrier function of host cells based on different FOS and *L. plantarum* treatments, immunofluorescent staining images or the related gene expression of the barrier protein are needed. Moreover, Caco-2 and BeWo cells are in vitro models, in vivo validation needs to be performed in the future. Based on these in vitro results, it could be inferred that FOS and *L. plantarum* might be useful for reducing the virulence of *L. monocytogenes*.

*L. monocytogenes* infection involves many virulence factors [72]. It can be internalized into host cells under the regulation of *inlA* and *inlB* [50]. Our data on the transcriptional profile of *L. monocytogenes* indicated that after 14 days at 10 °C and 40 h at 25 °C, *inlA* and *inlB* genes were down-regulated by co-treatment with *L. plantarum* and FOS, suggesting that the internalization of *L. monocytogenes* into host cells was reduced. This also explained the possible reasons for the decrease of the invasion ability of *L. monocytogenes* to Caco-2 and BeWo cells after the co-treatment of *L. plantarum* and FOS. After *L. monocytogenes* enters into the host cell, *hly* helps to accelerate *L. monocytogenes* escaping from phagocytic cells [75]. Tanner et al. reported that *Bifidobacterium thermophilum* RBL67 decreased the expression of the *hly* gene in *L. monocytogenes* [76]. Like the previous results, the *L. plantarum* and FOS treatment also significantly reduced *hly* gene expression. Moreover, we further evaluated the relative expression of *prfA*, which could activate the expression of *inlA*, *inlB*, *hly*, and other essential virulence genes in the LIPI1 cluster gene products and major internalins [77]. Our previous study showed that the *prfA* gene expression was reduced using *L. plantarum* combined with CO_2_ treatment [15]. Similarly, FOS, *L. plantarum*, and their combined treatment also down-regulated the expression of the *prfA* gene. Another virulence factor, *actA*, plays an important role in the capacity of the *L. monocytogenes* to polymerize actin and spread from cell to cell [52]. An important role of *inlA*, *inlB*, and *actA* in the process of invasion through the placental syncytiotrophoblast layer has been shown in human placental explants and animal models [36]. Thus, it is suggested that after the treatment of *L. plantarum* and FOS, the down-regulation of *inlA*, *inlB*, and *actA* expression may contribute to the reduction of *L. monocytogenes* adhesion and invasion to BeWo cells. Furthermore, *sigB* plays a vital role in the infectious cycle of *L. monocytogenes*, including regulating *inlA* and *inlB*, and down-regulating flagellum production before internalization [78]. Based on these discussions, under the FOS or *L. plantarum* treatment, the inhibition of the invasion process of *L. monocytogenes* to Caco-2 and BeWo cells was attributed to the down-regulation of *inlA*, *inlB*, *sigB*, *hly*, *actA*, and *PrfA* expression.

## 5. Conclusions

This study demonstrated that the presence of *L. plantarum* and FOS in the medium could reduce the pathogenic potential of *L. monocytogenes* by inhibiting the growth, decreasing the capability to adhesion and invasion of Caco-2 and BeWo cells, and down-regulating the virulence genes expression. The strain of *L. plantarum* can be used as a protective culture to inhibit *L. monocytogenes*. *L. plantarum* combined with FOS had a stronger ability than *L. plantarum* or FOS individually to reduce the pathogenic potential of *L. monocytogenes*. Specifically, *L. plantarum* combined with 2% or 4% FOS had the most obvious inhibitory effect on *L. monocytogenes*. However, there are some future directions to use and apply this technique in food to know the real food substrate as well to design an in vivo study to validate the current findings related to virulent genes.

## Figures and Tables

**Table 1:** The primer sequences used for RT-qPCR analyses [55].

Gene	Primer Sequences	Length (bp)
*rpoB*	F: TCGTCGTCTTCGTTCTGTTGR: GTTCGCCAAGTGGATTTGTT	221
*inlA*	F: ATAGGCACATTGGCGAGTTT	160
	R: GTGCGGTTAAACCTGCTAGG	
*inlB*	F: AAGCAMGATTTCATGGGAGAGT	78
	R: TTACCGTTCCATCAACATCATAACTT	
*hly*	F: CTTTTAACCGGGAAACACCA	302
	R: TCTTGCGTTACCTGGCAAA	
*actA*	F: CGGGTAAATGGGTACGTGAT	85
	R: TGGTCAATTAACCCTGCACTT	
*prfA*	F: CGGGAAGCTTGGCTCTATTTG	150
	R: GCTAACAGCTGAGCTATGTGC	
*sigB*	F: TCATCGGTGTCACGGAAGAA	310
	R: TGACGTTGGATTCTAGACAC	

## Data Availability

All data related to the research are presented in the article.
